# Time Trends in Racial/Ethnic Differences in COVID-19 Infection and Mortality

**DOI:** 10.3390/ijerph18094848

**Published:** 2021-05-01

**Authors:** Michelle S. Wong, Taona P. Haderlein, Anita H. Yuan, Ernest Moy, Kenneth T. Jones, Donna L. Washington

**Affiliations:** 1 VA HSR&D Center for the Study of Healthcare Innovation, Implementation & Policy (CSHIIP), VA Greater Los Angeles Healthcare System 11301 Wilshire Blvd, Los Angeles, CA 90073, USA; Taona.Haderlein@va.gov (T.P.H.); Anita.Yuan@va.gov (A.H.Y.); Donna.Washington@va.gov (D.L.W.); 2 VHA Office of Health Equity, 810 Vermont Ave NW, Washington, DC 20420, USA; Ernest.Moy@va.gov (E.M.); Kenneth.Jones8@va.gov (K.T.J.); 3 Department of Medicine, Division of General Internal Medicine and Health Services Research, University of California Los Angeles Geffen School of Medicine, 1100 Glendon Ave STE 850, Los Angeles, CA 90024, USA

**Keywords:** veterans, COVID-19, racial/ethnic disparities

## Abstract

Studies documenting coronavirus disease 2019 (COVID-19) racial/ethnic disparities in the United States were limited to data from the initial few months of the pandemic, did not account for changes over time, and focused primarily on Black and Hispanic minority groups. To fill these gaps, we examined time trends in racial/ethnic disparities in COVID-19 infection and mortality. We used the Veteran Health Administration’s (VHA) national database of veteran COVID-19 infections over three time periods: 3/1/2020–5/31/2020 (spring); 6/1/2020–8/31/2020 (summer); and 9/1/2020–11/25/2020 (fall). We calculated COVID-19 infection and mortality predicted probabilities from logistic regression models that included time period-by-race/ethnicity interaction terms, and controlled for age, gender, and prior diagnosis of CDC risk factors. Racial/ethnic groups at higher risk for COVID-19 infection and mortality changed over time. American Indian/Alaskan Natives (AI/AN), Blacks, Hispanics, and Native Hawaiians/Other Pacific Islanders experienced higher COVID-19 infections compared to Whites during the summertime. There were mortality disparities for Blacks in springtime, and AI/ANs, Asians, and Hispanics in summertime. Policy makers should consider the dynamic nature of racial/ethnic disparities as the pandemic evolves, and potential effects of risk mitigation and other (e.g., economic) policies on these disparities. Researchers should consider how trends in disparities change over time in other samples.

## 1. Introduction

A recent systematic review of the novel coronavirus disease 2019 (COVID-19) racial/ethnic disparities in the United States found strong evidence that non-Hispanic Black and Hispanic individuals experienced higher infection rates and excess mortality due to COVID-19 compared to non-Hispanic Whites [1]. Though American Indian and Alaskan Native (AI/AN) and Native Hawaiian/Other Pacific Islander (NHOPI) individuals also experienced excess mortality, the strength of evidence was weaker due to limited inclusion in studies [1]. Additionally, studies have found that Asian individuals had similar or higher rates of infection compared to non-Hispanic White individuals, but the strength of the evidence was also low because few studies included Asian individuals or disaggregated them from NHOPI individuals [1].

Another significant limitation of existing studies is that these studies were cross-sectional analyses using data from the early months of the pandemic (March to June 2020) [1]. However, the pandemic has changed significantly in ways that might affect trends in racial/ethnic disparities. Some of these changes may potentially decrease racial/ethnic disparities. First, there is greater awareness of COVID-19 racial/ethnic disparities [1]. This has led to national, state, and local health agency efforts to address these disparities [2,3]. For example, the Centers for Disease Control and Prevention (CDC) is partnering with community organizations to increase COVID-19 testing, conduct contact tracing in communities of color that are disproportionately affected by COVID-19, and culturally tailor information about COVID-19 to diverse populations (e.g., making this information available in multiple languages) [3]. Healthcare systems and hospitals have engaged community health workers, who are trusted members of local communities with shared lived experience, to bridge medical distrust among racial/ethnic minority individuals. Community health workers have been instrumental in providing reliable health information, combatting misinformation, and helping racial/ethnic minority individuals navigate the healthcare system and social support resources [4,5]. Moreover, while COVID-19 infections were initially concentrated in urban areas, which typically had larger racial/ethnic minority populations, the pandemic is now widespread in the United States [6]. Hot spots emerged later on in the pandemic in rural regions of the United States that are predominately non-Hispanic White.

However, it is unclear how other changes during the pandemic have affected trends in racial/ethnic disparities. Improved treatments have increased COVID-19 survival [7], but the high cost of some treatments, such as monoclonal antibodies, has limited their availability, making it potentially inaccessible to those who are at highest risk of COVID-19 infection or serious outcomes [8]. One study found that non-Hispanic Black patients with invasive cancer and COVID-19 were less likely to receive remdesivir, an antiviral agent that has been shown to reduce COVID-19 mortality, compared to non-Hispanic White patients [9]. Social distancing policies, such as stay-at-home orders and mandated business closures, have successfully slowed COVID-19 transmission [10]. However, racial/ethnic minorities, especially Black and Hispanic individuals, who are overrepresented among essential workers [11], may not be able to adhere to stay-at-home orders [12]. States and local governments have periodically enacted social distancing policies as COVID-19 cases increased, but it is possible that racial/ethnic disparities may increase each time these policies are in place.

Given the evolution of the pandemic, it is unknown how racial/ethnic disparities in COVID-19 have changed over time, especially in racial/ethnic groups that have been less well studied. To address these gaps, we examined racial/ethnic differences in COVID-19 infection and mortality over three time periods from 3/1/2020–11/25/2020 in a national and racial/ethnically diverse sample of veterans. We hypothesized that racial/ethnic disparities in COVID-19 infection and mortality would change over time, with disparities decreasing over time. Our findings have implications for potential infection control strategies for future surges in the COVID-19 pandemic, and also highlight the dynamic nature of racial/ethnic disparities in emergency crisis situations.

## 2. Materials & Methods

### 2.1. Data and Sample

Data came from Veterans Health Administration’s (VHA) national database of veterans who were evaluated for potential COVID-19 infection at any VHA facility. Our sample included 705,715 veterans who received COVID-19 polymerase chain reaction tests of nasopharyngeal swab specimens between 3/1/2020–11/25/2020, excluding veteran employees.

### 2.2. Measures

We examined two dichotomous dependent variables: testing positive for COVID-19 and mortality among those who tested positive. Our main independent variable was a categorical indicator of veteran race/ethnicity, ascertained from Veterans Affairs (VA) electronic health records: AI/AN, Asian, non-Hispanic Black, Hispanic, NHOPI, and non-Hispanic White (reference). To examine changes over time, we considered time (based on the date when the COVID-19 test was conducted) as an effect modifier with the following three time periods: 3/1/2020–5/31/2020 (spring); 6/1/2020–8/31/2020 (summer); and 9/1/2020–11/25/2020 (fall). These time periods of approximately three-month intervals corresponded to peaks and troughs in U.S. COVID-19 case counts [13].

We controlled for age (categorical: <60 years, 60–64 years, 65–69 years, 70–74 years, 75–79 years, or 80+ years), gender (dichotomous: male or female), and whether or not veterans had prior diagnoses of comorbidities listed by the Center for Disease Control and Prevention (CDC) as risk factors for severe COVID-19 (dichotomous: yes or no for each of the following conditions—chronic kidney disease stage-5/end-stage renal disease; chronic pulmonary disease; diabetes; heart disease; immunocompromised state; liver disease; obesity; and asthma) [14,15,16,17].

### 2.3. Analysis

We calculated overall descriptive statistics for the sample that received a COVID-19 test and for those that tested positive. For both samples (received a COVID-19 test and tested positive), we next calculated descriptive statistics stratified by racial/ethnic group and used a chi-squared test to determine whether there were statistically significant differences in covariates by race/ethnicity. We also calculated COVID-19 test positivity and mortality proportions for each racial/ethnic group by time period and overall. We then used the independent t-test to compare bivariate differences in COVID-19 infection and mortality for each racial/ethnic minority group relative to non-Hispanic Whites (reference) for each time period. We fit logistic regression models with an interaction term between race/ethnicity and time period to determine racial/ethnic differences over time for COVID-19 infection among veterans tested for COVID-19, and mortality among those testing positive for COVID-19. We adjusted for control variables described above and clustered standard errors by VHA facility to account for facility-level and regional variation in COVID-19 infection and testing. In addition to presenting adjusted odds ratios, we expressed the effects of race/ethnicity as adjusted predicted probabilities of COVID-19 infection and mortality for each racial/ethnic group, which we calculated from the adjusted logistic regression models using the margins post estimation command. The predicted probabilities reflect adjustment to average values on measures of confounders. [18] To determine statistically significant racial/ethnic differences within each time period, we calculated linear combinations of odds ratios for each racial/ethnic group relative to non-Hispanic Whites by time period. We determined statistical significance at *p* < 0.05. Analyses were conducted in Stata version 15.1 (College Station, TX, USA). VA Greater Los Angeles Healthcare System Institutional Review Board determined this to be a non-research activity.

## 3. Results

Table 1 presents characteristics of the full sample of veterans who were evaluated for COVID-19 at VHA and among those who tested positive. Among those who were evaluated, 62% were non-Hispanic White, 22% were non-Hispanic Black, and 9% were Hispanic. Those who received a COVID-19 test were more likely to be less than the age of 60 (39%). The most common comorbidities in this sample included obesity (50%), heart disease (36%), or type 2 diabetes (34%). More than half of the COVID-19 tests were administered in the fall.

Among veterans who tested positive (*n* = 83,542), 56% were non-Hispanic White, approximately one quarter were non-Hispanic Black, and 11% were Hispanic. AI/AN, Asian, and NHOPI veterans each comprised less than 1% of the sample of those testing positive. Those who tested positive for COVID-19 were also more likely to be less than 60 years of age. Nearly 60% of the sample had obesity, followed by type 2 diabetes (36%), and heart disease (32%).

There were racial/ethnic differences in all studied variables among both samples of veterans who received a COVID-19 test and who tested positive for COVID-19 (Table 2). Non-Hispanic White veterans who received a COVID-19 test were more likely to be older, male, and received a COVID-19 test in the fall, compared to other racial/ethnic groups. Non-Hispanic Whites had the smallest proportion of veterans with asthma and chronic kidney disease, while Asians had the smallest proportion with chronic pulmonary disease, type 2 diabetes, heart disease, immunocompromisation, liver disease, and obesity. In contrast, non-Hispanic Whites had the largest proportion with chronic pulmonary disease and heart disease. Non-Hispanic Blacks were most likely to have chronic kidney disease, type 2 diabetes, or be immunocompromised. Hispanics were more likely to have liver disease. More than half of non-Hispanic Whites, AI/ANs, Hispanics, and NHOPI veterans in the sample were obese. Patterns in racial/ethnic differences among veterans who tested positive for COVID-19 were generally similar to the full sample of those who were tested, with a few exceptions. Asians had the smallest proportion of veterans with chronic kidney disease, while NHOPIs had the smallest proportion with liver disease.

Twelve percent of the sample tested positive for COVID-19 across the entire time period. Table 3 presents unadjusted proportion of COVID-19 test positivity and mortality for each racial/ethnic group by time period and overall, as well as bivariate comparisons for each racial/ethnic minority group relative to non-Hispanic White veterans (reference). In the spring, the COVID-19 test positivity proportion was highest among non-Hispanic Black veterans (22%), followed by Hispanic veterans (16%). However, in the summer, Hispanic veterans were most likely to test positive (19%). During this period, a larger proportion of AI/AN, non-Hispanic Black, Hispanics, and NHOPI veterans tested positive for COVID-19 compared to non-Hispanic White veterans. In the fall, AI/AN veterans were most likely to test positive (15%), followed by Hispanic veterans (13%), and NHOPI veterans (13%). Compared to non-Hispanic White veterans, COVID-19 infection proportions remained higher among AI/AN and Hispanic veterans in the fall, but were lower among non-Hispanic Blacks and Asian veterans. The unadjusted case fatality rate decreased over time from 13.8% (spring) to 5.5% (summer), then to 2.5% (fall). In the spring and summer, mortality proportion was highest among AI/AN veterans (17% and 10%, respectively), followed by non-Hispanic White veterans (16% and 6.5%, respectively). In the fall, non-Hispanic White veterans had the highest mortality proportion (2.9%), followed closely by NHOPI (2.8%) and AI/AN veterans (2.7%). Across all three time periods, non-Hispanic Black and Hispanic veterans had a statistically significantly unadjusted lower mortality proportion than non-Hispanic White veterans.

**Figure 1:**
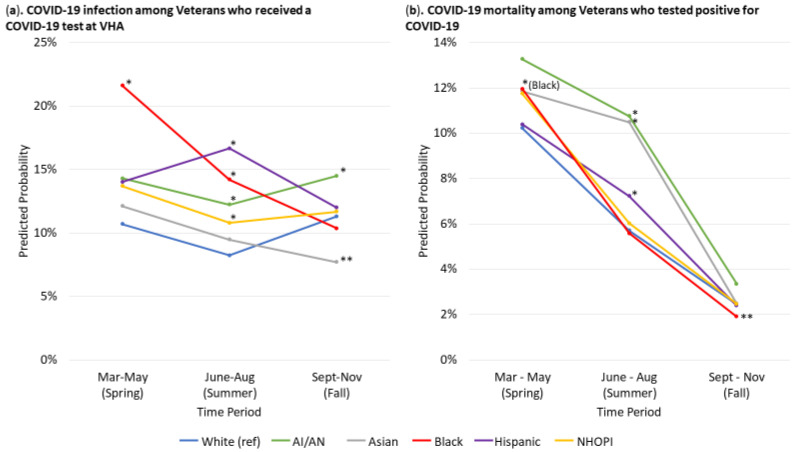
Adjusted predicted probability of COVID-19 infection and mortality by race/ethnicity over time. (**a**) presents the adjusted predicted probability of COVID-19 infection by race/ethnicity over time among Veterans who received a COVID1-9 test at VHA. (**b**) presents the adjusted predicted probability of COVID-19 mortality by race/ethnicity over time among Veterans who tested positive for COVID-19. Note: * denotes statistically significant differences at *p* < 0.05 that favor the reference group of non-Hispanic White veterans (i.e., disparities); ** denotes statistically significant differences at *p* < 0.05 that favor the racial/ethnic minority group versus the reference group of non-Hispanic White veterans. Models adjusted for age, sex, and prior diagnoses of CDC-identified risk factors (chronic kidney disease stage-5/end-stage renal disease; chronic pulmonary disease; diabetes; heart disease; immunocompromised state; liver disease; obesity; and asthma). Models included multi-race individuals and those with missing or unknown race/ethnicity, but results are not shown.

Table 4 presents adjusted odds ratios for COVID-19 infection among veterans who received a COVID-19 test and mortality among veterans who tested positive for COVID-19 by time period. Figure 1 presents adjusted predicted probabilities for COVID-19 infection and mortality, calculated from adjusted logistic regression models, for each racial/ethnic group by time period.

### 3.1. COVID-19 Infection

The predicted probability of COVID-19 infection, adjusting for age, gender, and CDC-risk factors was higher among non-Hispanic Black veterans (21.6%, 95% confidence interval [CI]: 18.2%, 25.0%) compared to non-Hispanic White veterans (10.7%, 95% CI: 9.1%, 12.3%) in springtime, and similar between all other groups (12%–14%) and non-Hispanic White veterans (Figure 1a). During summer, predicted probabilities were higher in AI/AN (12.2%, 95% CI: 10.3%, 14.2%), non-Hispanic Black (14.2%, 95% CI: 12.5%, 15.9%), Hispanic (16.7%, 95% CI: 10.9%, 22.5%), and NHOPI veterans (10.8%, 95% CI: 8.6%, 13.0%), and similar in Asian veterans (9.5%, 95% CI: 7.1%, 16.8%), compared to non-Hispanic White veterans (8.2%, 95% CI: 7.3%, 9.2%). In the fall, only AI/AN veterans (14.5%, 95% CI: 11.7%, 17.3%) continued to have a higher predicted probability of COVID-19 infection compared to non-Hispanic White veterans (11.3%, 95% CI: 10.1%, 12.5%). In contrast, Asian veterans (7.7%, 95% CI: 5.4%, 10.0%) had a lower predicted probability of COVID-19 infection relative to non-Hispanic White veterans.

### 3.2. Mortality

The adjusted case fatality rate decreased over time for all racial/ethnic groups. With adjustment for age, gender, and prior diagnoses of CDC-risk factors, initially, non-Hispanic Black veterans had a higher adjusted case fatality rate (12.0%, 95% CI: 10.5%, 13.4%) and other racial/ethnic groups had statistically similar case fatality rates (10–13%) as non-Hispanic White veterans (10.2%, 95% CI: 9.3%, 11.2%) (Figure 1b). The decline in the adjusted case fatality rate from spring to summer was lower for AI/AN (10.7%, 95% CI: 5.7%, 15.8%), Asian (10.5%, 95% CI:5.2%, 15.7%), and Hispanic veterans (7.2%, 95% CI: 5.8%, 8.7%), and these groups had greater case fatality than non-Hispanic White veterans (5.7%, 95% CI: 5.2%, 6.2%). The adjusted case fatality rate declined further for all groups from summer to fall. The overall spring-to-fall decline was greatest for non-Hispanic Black veterans, and in the fall, non-Hispanic Black veterans (1.9%, 95% CI: 1.4%, 2.4%) had a lower adjusted case fatality rate compared to non-Hispanic White veterans (2.5%, 95% CI: 2.2%, 2.7%), whereas other groups (2.4–3.3%) were similar to non-Hispanic White veterans.

## 4. Discussion

Although racial/ethnic disparities in COVID-19 infection and outcomes for non-Hispanic Black and Hispanic individuals were well documented at the beginning of the pandemic [1], we found that the racial/ethnic groups at highest risk for COVID-19 infection and mortality changed over time in this national sample of veterans who received a COVID-19 test at a VHA facility. More racial/ethnic groups experienced disparities during the summer relative to non-Hispanic White veterans. During the summer, COVID-19 infections were higher among AI/AN, non-Hispanic Black, Hispanic, and NHOPI veterans, and mortality was higher among AI/AN, Asian, and Hispanic veterans. AI/AN veterans continued to experience COVID-19 infection disparities in the fall.

Our findings are generally consistent with evidence of higher COVID-19 infection among Hispanics and non-Hispanic Black individuals [1,19]. However, we are encouraged to see that among veterans, COVID-19 infections decreased for non-Hispanic Blacks over time. VA and general messaging promoting social distancing that target non-Hispanic Black communities may have helped to reduce COVID-19 infections. Use of masks and facial coverings by the general public may have also reduced COVID-19 transmission to essential workers, who are more likely to be racial/ethnic minorities.

There is strong evidence in the general U.S. population of higher COVID-19-related mortality among non-Hispanic Blacks [1], but we only found Black–White mortality disparities in this sample of veterans early in the pandemic. This differs from an early report of VHA users that was conducted using data from 2/8/2020–5/4/2020 [19], that had less power to detect racial/ethnic differences, and that did not identify racial/ethnic differences. Understanding of COVID-19 risk factors have changed over time [17,20], and our analysis also reflects current CDC identified risk factors. VHA’s success in reducing health disparities in outcomes and care for non-Hispanic Black veterans, including mortality [21,22], may also have contributed to smaller Black–White mortality differences among veterans who tested positive for COVID-19 in this sample compared to the general U.S. population. Unlike other healthcare systems, VA has reduced financial barriers to care, has greater knowledge of patients’ comorbidities and medications as a result of established relationship with patients, and collects robust race/ethnicity data to monitor and mitigate disparities [23,24]. That we found changes in infection and mortality disparity trends for non-Hispanic Black and Hispanic patients as the pandemic evolved highlights the need to also examine changes over time in other samples for these two racial/ethnic groups.

Few studies have included the smaller racial/ethnic minority groups. Our study takes advantage of VHA’s racially/ethnically diverse patient population to examine disparities among AI/AN, NHOPI, and Asian individuals. Our study adds to growing evidence of COVID-19’s devastating effects on AI/AN individuals [25]. We found disparities even among AI/AN individuals who accessed healthcare, and that infections have not decreased over time. It is imperative to understand other factors that contribute to these disparities. AI/AN individuals have long experienced historical and political oppression, systemic racism, and violence, all of which made them especially vulnerable to the COVID-19 pandemic [26]. As a result, they face unique social determinants, such as poverty and substandard housing conditions, that may explain COVID-19’s disproportion effect on AI/AN individuals, especially those residing on federally recognized tribal lands [27,28]. NHOPI veterans in our sample had higher risk of COVID-19 infection over the summer. Many Pacific Islander individuals work in healthcare [29], which may increase their risk of COVID-19 infection. We also found higher mortality over the summer in Asian veterans, whereas some studies, conducted with data from the early months of the pandemic, suggested that Asian individuals in the general U.S. population had similar or lower COVID-19-related mortality than non-Hispanic White individuals [1]. Further disaggregating Asian veteran VHA users may yield additional insight into this disparity. It is also possible that trends in COVID-19 infections among Asian individuals have changed over time, but more evidence in other samples of the U.S. general population is needed to confirm this. Our results underscore the importance of also monitoring COVID-19’s impact on other racial/ethnic groups besides non-Hispanic Black and Hispanic individuals, that comprise smaller proportions of the U.S. population.

Although mortality decreased over time, more racial/ethnic minority groups experienced infection and mortality disparities over the summer. Increased infections may have resulted from reopening businesses, leading to increased workplace exposure, particularly for racial/ethnic minorities who are more likely to be employed as essential or healthcare workers [11]. It is possible that improvements in COVID-19 treatment may have initially exacerbated disparities, if VA facilities serving more racial/ethnic minority patients were slower in adopting new treatments. Future studies can examine how trends in racial/ethnic disparities correspond to state and local government mandates for businesses to close and reopen, changes to social distancing policies, whether patients of different race/ethnicities experienced differential access to COVID-19 treatments, and how these differences affected racial/ethnic disparities.

Our study had several limitations. First, we conducted this evaluation in a sample that had access to and received care from VHA’s integrated healthcare system. Our results may not be generalizable to other populations that do not have access to healthcare or to populations served by other healthcare systems. Racial/ethnic disparities found in our sample may be smaller than differences in other samples because limited healthcare access and worse healthcare quality for racial/ethnic minorities was hypothesized as a contributor to disparities in COVID-19 [30]. Additionally, while there was geographic variation in the pandemic’s hot spots, social distancing policies, and healthcare systems’ testing practices, and availability and adoption of treatments, our analysis did not consider differences by geography over time. We also did not account for social determinants of health, such as employment and job type, which have also contributed to racial/ethnic disparities in COVID-19 [30]. Finally, mortality among AI/AN veterans may be overrepresented in our sample, because VA, as part of its fourth mission, supported Indian Health Service clinics in Indian Country to provide care to sicker COVID-19 patients [31], including AI/AN veterans. However, our study also had several strengths, including use of a national and racially/ethnically diverse sample and data collected throughout the course of the pandemic, which allowed us to consider trends over time.

## 5. Conclusions

Patterns of racial/ethnic COVID-19 disparities in infection and mortality have changed over time as social policies have altered exposure, and clinical care have evolved. Racial/ethnic minority groups may be at higher risk for infection as businesses reopen, given their over-representation among essential workers. VHA should continue to monitor racial/ethnic differences as the pandemic progresses, especially during surges, and tailor risk mitigation messaging to racial/ethnic groups at higher risk for COVID-19. Health disparities researchers should consider examining changes in racial/ethnic disparity trends over time in other samples, related to changing social policies.

Existing studies have examined racial/ethnic disparities in COVID-19 as a static phenomenon, and we believe that we are among the first to consider how these trends have changed over time. Our findings underscore the fact that for emergent crises, such as the COVID-19 pandemic, disparities are often dynamic. Some risk factors, such as advanced age, contributing to mortality risk [32] are persistent, although effect sizes may change. Other disparities, such as by race/ethnicity, may increase, disappear, or even reverse over time, as behavior and policies evolve with increasing knowledge of the crisis. Thus, disparities should be interpreted in the context of time.

## Figures and Tables

**Table 1:** Sample characteristics for veterans who received a COVID-19 test at a Veterans Health Administration (VHA) facility and for veterans who tested positive for COVID-19.

	Tested (*n* = 705,715)	Tested Positive (*n* = 83,542)
Race/Ethnicity, %		
Non-Hispanic White	61.8	56.1
AI/AN	0.7	0.8
Asian	1.1	0.8
Non-Hispanic Black	22.1	25.2
Hispanic	8.5	10.9
NHOPI	0.7	0.7
Age, %		
<60 years	39	43.7
60–64 years	11.7	10.1
65–69 years	12	10.2
70–74 years	19.4	17.1
75–79 years	9	8.3
80+ years	8.9	10.6
Gender, %		
Female	10.9	10.1
Male	89.1	89.9
CDC Co-morbidities, %		
Asthma	7.1	6.7
Chronic Kidney Disease	2	1.9
Chronic pulmonary disease	25	20.6
Types 2 Diabetes	33.5	35.6
Heart Disease	35.9	32.4
Immunocompromised	9.7	7.6
Liver Disease	10.5	8
Obesity	50.2	58
Time Period of Visit, %		
Spring (3/1/20–5/31/20)	8.1	13.5
Summer (6/1/20–8/31/20)	36.2	33.1
Fall (9/1/20–11/25/20)	55.7	53.4

Note: Proportion for all covariates presented are column percentages for the sample of veterans who received a COVID-19 test at a VHA facility and for the subsample who tested positive for COVID-19. Table does not include multi-racial individuals (*n* = 6354) or individuals with unknown or missing race/ethnicity (*n* = 30,157) who comprise approximately 5% of the sample.

**Table 2:** Sample characteristics of veterans who received a COVID-19 test and tested positive, stratified by race/ethnicity.

	Race/Ethnicity						*p*-Value
	White (*n* = 436,022)	AI/AN (*n* = 4860)	Asian (*n* = 7635)	Black (*n* = 155,787)	Hispanic (*n* = 59,740)	NHOPI (*n* = 5159)	
*Tested*							
Age categories, %							
<60 years	34.6	44.3	66.2	42.9	54.2	46	<0.001
60–64 years	10.2	12.8	7.6	17	9.8	11.1	
65–69 years	11.7	11.9	8.3	14.4	9.3	12	
70–74 years	22.5	18.1	8.6	14.1	13.3	16.5	
75–79 years	10.7	7	4.4	6.1	5.9	7.5	
80+ years	10.3	5.9	4.9	5.6	7.5	6.9	
Gender, %							
Female	9	15.2	14.2	15.4	11.5	13.7	<0.001
Male	91	84.8	85.8	84.6	88.5	86.3	
CDC comorbidities, %							
Asthma	6.5	7.4	8.8	8.1	8.8	7.8	<0.001
Chronic kidney disease	1.3	2.1	2.1	3.8	2.1	2.4	<0.001
Chronic pulmonary disease	27.5	24.1	13.9	22.4	16.9	21	<0.001
Type 2 diabetes	32.4	36.6	28.7	37.5	32.9	35.5	<0.001
Heart disease	39.8	31.5	19.4	31.3	24.9	31.4	<0.001
Immunocompromised	9.5	8.4	5.5	11.5	8	8.3	<0.001
Liver disease	10.1	11.5	8.7	11.7	12.1	9.5	<0.001
Obesity	50.4	53	34.3	49.9	52.4	52.6	<0.001
Time Period, %							
Spring (3/1/2020–5/31/2020)	8	9.2	7.2	9	7.3	7.2	<0.001
Summer (6/1/2020–8/31/2020)	35.8	36.4	38.4	36.5	36.9	37.7	
Fall (9/1/2020–11/25/2020)	56.2	54.4	54.4	54.5	55.8	55.1	
*Tested positive*							
Age categories, %							
<60 years	37	47.6	72.5	48.3	63.7	48.4	<0.001
60–64 years	8.3	10.8	7.3	15	8.5	10	
65–69 years	9.9	12.1	7.1	12.4	7.3	12.8	
70–74 years	20.8	17.8	6.4	12.6	10.4	15.1	
75–79 years	10.5	5.3	2.2	5.5	4.3	5.9	
80+ years	13.4	6.3	4.5	6.4	5.8	7.9	
Gender, %							
Female	8	15	13.7	14.7	10.3	11.1	<0.001
Male	92	85	86.3	85.3	89.7	88.9	
CDC comorbidities, %							
Asthma	6.2	7.7	7.9	7.7	7.2	7.3	<0.001
Chronic kidney disease	1.2	2	0.9	3.6	1.5	2.4	<0.001
Chronic pulmonary disease	23	20.9	12.7	19.2	12.8	16.5	<0.001
Type 2 diabetes	34.6	36.8	25	40.2	32.5	39.9	<0.001
Heart disease	37	28.3	19	28.6	19.7	27.8	<0.001
Immunocompromised	7.1	6.2	5.1	9.9	5.7	5.5	<0.001
Liver disease	7.3	8.7	8.8	9	9.8	5.3	<0.001
Obesity	57.3	59	46.9	58.7	61.6	59.4	<0.001
Time Period, %							
Spring (3/1/2020–5/31/2020)	11.1	11.5	14.1	20.5	10.7	10.6	<0.001
Summer (6/1/2020–8/31/2020)	28.5	33.2	37.8	38.6	43.2	34.4	
Fall (9/1/2020–11/25/2020)	60.4	55.3	48.2	40.9	46.1	55	

Note: Proportions presented are column proportions within each racial/ethnic group. *p*-values calculated from chi-squared test to compare proportions by race/ethnicity. Table does not include multi-racial individuals (*n* = 6354) or individuals with unknown or missing race/ethnicity (*n* = 30,157), who collectively comprise approximately 5% of the sample.

**Table 3:** Unadjusted COVID-19 infection and mortality proportions by race/ethnicity for each time period from 3/1/2020–11/25/2020.

	COVID-19 Infection Proportion				Mortality Proportion			
	Time Period			Total	Time Period			Total
	Spring 3/1/20–5/31/20	Summer 6/1/20–8/31/20	Fall 9/1/20–11/25/20		Spring 3/1/20–5/31/20	Summer 6/1/20–8/31/20	Fall 9/1/20–11/25/20	
Non-Hispanic White (ref) (*n* = 436,023)	11.6	8.9	12.0	10.8	16.3	6.5	2.9	5.4
AI/AN (*n* = 4860)	14.1	13.7 **	15.2 **	14.6 **	16.7	9.9 *	2.7	6.7
Asian (*n* = 7635)	13.1	9.4	8.5 **	9.2 **	7.8 *	4.4	1.1	3.3 *
Non-Hispanic Black (*n* = 155,740)	22.1 **	14.8 **	10.5 **	13.1 **	12.0 **	4.1 **	1.6 **	4.7 **
Hispanic (*n* = 59,740)	15.6 **	18.6 **	13.1 **	15.3 **	8.8 **	4.4 **	1.6 **	3.6 **
NHOPI (*n* = 5159)	13.6	11.5 **	12.6	12.2 *	10.2	4.5 *	2.8	4.2

Note: * denotes statistically significant bivariate difference from the non-Hispanic White veteran group (reference) at *p* < 0.05; ** denotes statistically significant bivariate difference from the non-Hispanic White veteran group (reference) at *p* < 0.001. Table does not include multi-racial individuals (*n* = 6354) or individuals with unknown or missing race/ethnicity (*n* = 30,157), who collectively comprise approximately 5% of the sample.

**Table 4:** Adjusted odds ratios of COVID-19 infection among veterans who were evaluated for COVID-19 at VHA and odds ratios of mortality among veterans who tested positive for COVID-19 by race/ethnicity over time.

	COVID-19 Infection Proportion			Mortality Proportion		
	Spring 3/1/20–5/31/20	Summer 6/1/20–8/31/20	Fall 9/1/20–11/25/20	Spring 3/1/20–5/31/20	Summer 6/1/20–8/31/20	Fall 9/1/20–11/25/20
Non-Hispanic White (ref) (*n* = 436,023)	1.00 (ref)	1.00 (ref)	1.00 (ref)	1.00 (ref)	1.00 (ref)	1.00 (ref)
AI/AN (*n* = 4860)	1.40 (0.90, 2.16)	1.56 (1.31, 1.85) *	1.33 (1.08, 1.64) *	1.41 (0.70, 2.82)	2.16 (1.17, 3.97) *	1.40 (0.70, 2.81)
Asian (*n* = 7635)	1.15 (0.76, 1.74)	1.17 (0.89, 1.53)	0.65 (0.48, 0.89) **	1.21 (0.57, 2.58)	2.09 (1.09, 4.01) *	1.01 (0.25, 4.09)
Non-Hispanic Black (*n* = 155,787)	2.32 (1.95, 2.76) *	1.85 (1.66, 2.07) *	0.91 (0.81, 1.01)	1.23 (1.04, 1.45) *	0.98 (0.84, 1.14)	0.77 (0.60, 0.98) **
Hispanic (*n* = 59,740)	1.37 (0.93, 2.02)	2.24 (1.51, 3.32) *	1.07 (0.86, 1.34)	1.03 (0.70, 1.49)	1.32 (1.06, 1.64) *	0.97 (0.69, 1.36)
NHOPI (*n* = 5159)	1.33 (0.93, 1.89)	1.35 (1.10, 1.66) *	1.04 (0.85, 1.27)	1.20 (0.41, 3.52)	1.07 (0.54, 2.09)	1.00 (0.46, 2.20)

Note: * denotes statistically significant differences at *p* < 0.05 that favor the reference group of non-Hispanic White veterans (i.e., disparities); ** denotes statistically significant differences at *p* < 0.05 that favor the racial/ethnic minority group versus the reference group of non-Hispanic White veterans. Model adjusted for age, sex, and prior diagnoses of CDC-identified risk factors (chronic kidney disease stage-5/end-stage renal disease; chronic pulmonary disease; diabetes; heart disease; immunocompromised state; liver disease; obesity; and asthma). Models included multi-race individuals and those with missing or unknown race/ethnicity, but results are not shown.

## Data Availability

The dataset used in this study cannot be shared publicly because it is derived from clinical operations (non-research) data.
